# A modern, flexible cloud-based database and computing service for real-time analysis of vehicle emissions data

**DOI:** 10.1007/s44212-024-00066-4

**Published:** 2025-01-08

**Authors:** Christopher E. Rushton, James E. Tate, Åke Sjödin

**Affiliations:** 1 Institute for Transport Studies, University of Leeds, Leeds, LS2 9JT UK; 2 IVL Swedish Environmental Research Institute LTD., PO Box 530 21, SE-400 14 Gothenburg, Sweden

**Keywords:** Vehicle emissions, Cloud computing, Remote sensing, Point sampling, Real driving emissions

## Abstract

In response to the demand for advanced tools in environmental monitoring and policy formulation, this work leverages modern software and big data technologies to enhance novel road transport emissions research. This is achieved by making data and analysis tools more widely available and customisable so users can tailor outputs to their requirements. Through the novel combination of vehicle emissions remote sensing and cloud computing methodologies, these developments aim to reduce the barriers to understanding real-driving emissions (RDE) across urban environments. The platform demonstrates the practical application of modern cloud-computing resources in overcoming the complex demands of air quality management and policy monitoring. This paper shows the potential of modern technological solutions to improve the accessibility of environmental data for policy-making and the broader pursuit of sustainable urban development. The web-application is publicly and freely available at https://cares-public-app.azurewebsites.net.

## Introduction

Outdoor and indoor air pollution are the two leading causes of economic disadvantage from environmental factors respectively (Lomborg, 2020; Stanaway et al., 2018). Transport, especially in urban environments, is a major contributor to outdoor air pollution (Colvile et al., 2001; O’Driscoll et al., 2018; Pastorello & Melios, 2016). The level of exposure to air pollution and its subsequent negative effects will only be exacerbated by the current global trend of urbanisation (Gu et al., 2021). As more people move to the urban environment there is an increased demand for transport services creating a vicious circle of poor public health. The increase in demand causes a non-linear increase in pollution because the capacity of road networks are largely fixed and the increased demand leads to more severe and frequent congestion, which produces more air pollution per vehicle than non-congested traffic (WHO, 2006). This is likely to lead to an increased burden on the people responsible for delivering the policies aimed at reducing the population’s exposure to air pollution. With the complexities of cost and expertise as well as constrained capacity for delivery, the challenge of effectively reducing air pollution in urban environments remains, despite advances in vehicle emissions controls and the ongoing electrification of the fleet. This study aims to demonstrate the potential of this technology to reduce the complexity and cost burden on air pollution policy makers by allowing them access to up-to-date data and data processing tools.

Air pollution is a significant contributing factor to many health problems (Breslow & Goldsmith, 1958) relating to both cardiovasular and respiratory system problems (Brunekreef & Holgate, 2002). Exposure to air pollution can result in a reduction in lung function amongst children (Gauderman et al., 2004), a reduction in cognitive ability and depression, affecting both children and adults (Fonken et al., 2011; Gatto et al., 2014; Power et al., 2011). The two major air pollutants associated with transport in urban environments are oxides of nitrogen ($NO_{x}$), and more pressingly nitrogen dioxide ($NO_{2}$), and fine particulate matter ($PM_{2.5}$). $NO_{2}$ is generally regarded as a major contributor to poor health outcomes across a range of different domains (COMEAP, 2015; EEA, 2008; IARC, 2013; Kampa & Castanas, 2008; WHO, 2013; Zhang et al., 2011). Exposure to *PM*2.5 is also increased in transport environments (Adams et al., 2001; Kinney et al., 2011) and the negative health impacts are significant (Yang et al., 2019).

Air pollution is a negative external cost of transportation (Ward et al., 2021), making policy interventions the most effective way to reduce its impact. Policies that mitigate air pollution or reduce public exposure to it are highly desirable from both health and economic perspectives. The historical failure of type approval legislation to reduce ambient air pollution levels (Boogaard et al., 2012; Carslaw et al., 2011b; Carslaw, 2005; Carslaw et al., 2011a; Holman et al., 2015) indicates the need for new methods, which are increasingly critical for the economic and social well-being of city dwellers. Several factors limit the usefulness of laboratory measurements for developing effective policies, including the disparity between type approval limits and real driving emissions (RDE) values, and the growing number of vehicles with deteriorated or manipulated emissions reduction systems (Pöhler et al., 2023), making in-situ RDE measurements essential. Additionally, the impact of deterioration on emissions over a vehicle’s lifespan is relatively poorly understood. Combined with the differences between urban duty-cycles and test procedures, this may significantly affect overall emissions.

The significant health impacts associated with poor air quality and increasing political awareness of these issues have led to a rise in studies, legislation, and schemes aimed at improving air quality. However, communicating the results of these studies and assessing the effectiveness of new schemes and legislation remains challenging. Efforts have been made to make publicly funded air pollution data more accessible to the general public. Data from the Automatic Urban and Rural Network (AURN) in the UK are available online (https://uk-air.defra.gov.uk); however, accessing the data directly can be challenging and often requires specialised software and expertise. The openAir (Carslaw & Ropkins, 2012), worldMet (Carslaw, 2023), and AQEval (Ropkins et al., n.a.) *R* packages have lowered the barrier to entry for analysis, but they still require specialised technical expertise, likely beyond the knowledge of most citizens.

The National Center for Environmental Information, in partnership with the National Oceanic and Atmospheric Administration (NCEI, NOAA), offers an online platform where users can view various environmental visualisations within a Geographical Information Systems (GIS) environment such as https://www.ncei.noaa.gov/maps-and-geospatial-products. The Real Urban Emissions Initiative (TRUE Initiative, https://www.trueinitiative.org), a partnership between the FIA Foundation and the International Council on Clean Transportation (ICCT), has integrated vehicle lookup forms into its website, allowing users to check the air quality impact of individual vehicles. OpenAQ (https://openaq.org) provides an open data source for a global network of air pollution monitors that is publicly accessible through an API. The *RTVEMVS* project (Ding et al., 2021) uses real-time traffic flow data and vehicle metadata to model the vehicle emissions of vehicles on the Chongqing network, but does not measure them directly. The new approach presented in this paper is the first to make real driving emission data publicly available as well as using an innovative document style data structure to facilitate instruments and campaigns with different data outputs being displayed consistently and coherently. This improves the comparability of these data sets and ease of interpretation for users with less capacity or knowledge to use modern data science tools. This, in turn, allows policy decisions to be made using a joined-up data-driven approach rather than a more speculative or intuitive approach, and will lead to more successful policy development in the future.

Optical remote sensing device (RSD) measurement campaigns have historically shed light on the real driving emissions (RDE’s) of in-situ vehicles, giving operators a clearer picture of the contribution of different vehicle types to the overall emissions inventory. The method has been particularly successful for gaseous species such as carbon monoxide (*CO*), hydrocarbons (*HC*) and oxides of nitrogen (*NO*, $NO_2$ and $NO_X$) (Popp et al., 1999; Stedman & Bishop, 1990; Stedman et al., 1994; Zhang et al., 1995, 1996). Optical RSD measurements have been taken across the United Kingdom, continental Europe and worldwide (Carslaw, 2005; Franco et al., 2013; Rushton et al., 2018, 2021; Tate, 2013b). There are currently two leading commercial providers of RSD measurements, OPUS and HEAT. More detailed descriptions of the OPUS and HEAT remote sensing methodologies can be found elsewhere in the literature (Dallmann et al., 2019; Ghaffarpasand et al., 2020; Ropkins et al., 2017). A method for point sampling (PS), distinct from the optical remote sensing methodology has also been developed. The point sampling methodology may reduce the cost of RDE measurements by using less expensive instruments that could be deployed in greater number across cities (Knoll et al., 2023). This may be done with a potential five-fold improvement for particulate matter emission data quality and without significant loss of gaseous species data quality, as proven for *NO* and $NO_2$. (Giechaskiel et al., 2014; Horbanski et al., 2019; Knoll et al., 2024; Schriefl et al., 2020). The PS methodology and the RS methodology use light-gate technology to capture the individual vehicle pass-throughs and an automatic number plate recognition (ANPR) system was used to attribute tailpipe emissions to a specific vehicle. The number plates are used as a reference for vehicle meta-data and then pseudonymised to comply with data protection regulations. Speed and acceleration characteristics are also captured using the light-gate to give some indication of vehicle dynamics and power requirements at the measurement site (Carslaw et al., 2013).

This paper introduces the data infrastructure that was delivered as part of the CARES project (Davison et al., 2022; Farren et al., 2023; Knoll et al., 2023; Pechout et al., 2022; Pöhler et al., 2023; Qiu & Borken-Kleefeld, 2022; Yang et al., 2022) and a parallel Chinese research project running in parallel to CARES (Hao et al., 2022a, b) showing how it can be applied in the real world. A full list of CARES deliverables may be found at https://cordis.europa.eu/project/id/814966/results. This paper discusses and demonstrates the potential for modern data handling and data science techniques to be used in conjunction with the latest developments in vehicle RDE measurements to lower the barrier to entry for people wishing to engage with existing campaigns or conduct their own campaigns. The CARES project has provided the opportunity to research and develop modern data-science solutions to collate, quality check, process and analyse vehicle remote emission sensing data. The project has also merged more conventional vehicle remote emission sensing measurements made by commercial suppliers with the emerging point sampling solution. The novel use of PS devices and a modern cloud-based data management and processing infrastructure was intended to reduce the barrier of entry for policy makers and reduce their reliance on external contractors to analyse their data.

## Data system development

### Data Collection

**Fig. 1:**
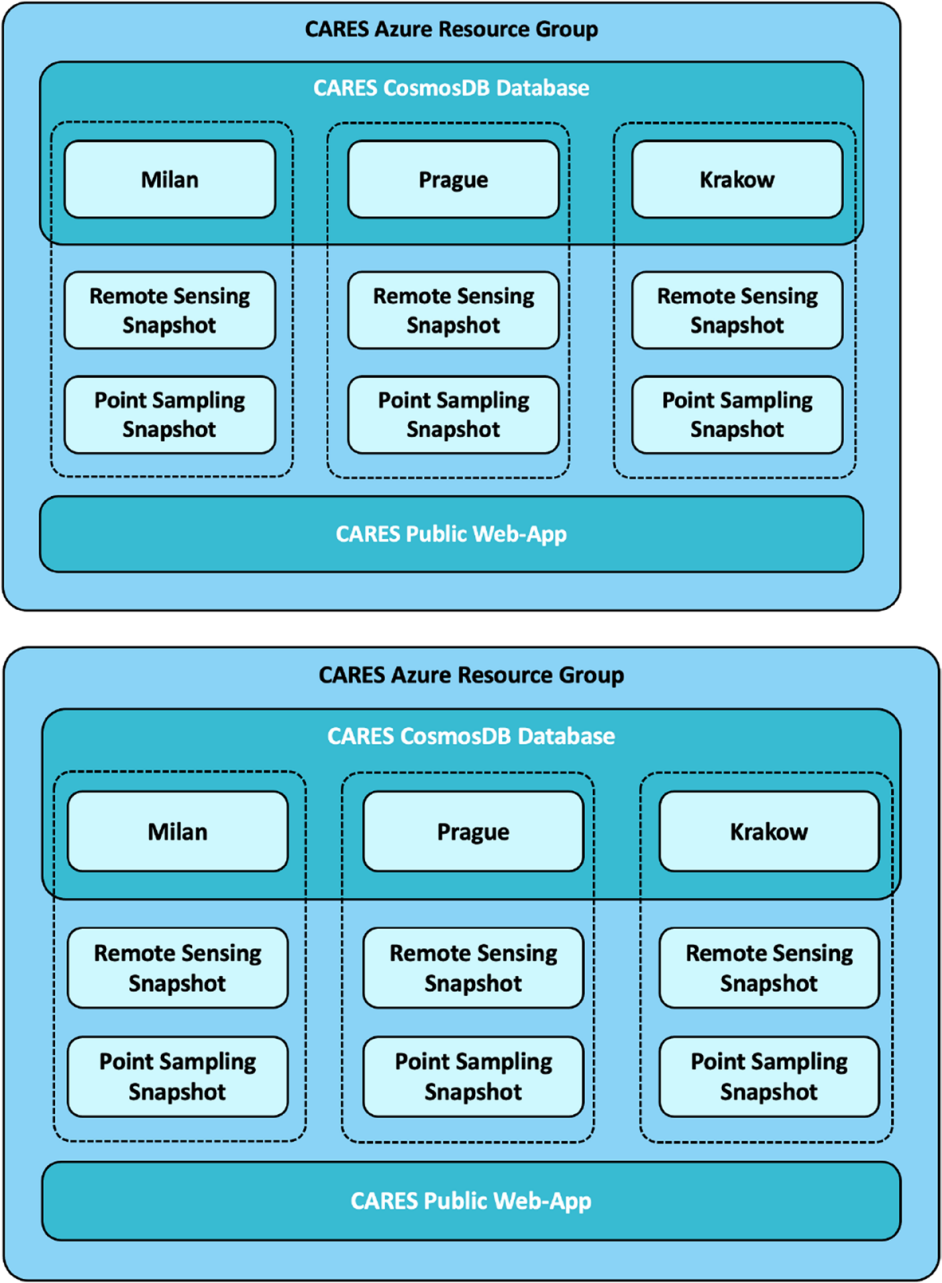
Schematic showing data infrastructure and the different data sets available for each city

The data collection for this project consisted of four separate campaigns. An instrument characterisation experiment was conducted at Lelystadt (NL), and three city experiments were respectively conducted in Milan (IT), Prague (CZ) and Krakow (PL). Each experiment consisted of a different set of sensors. The data collection and key results are detailed throughout the cited publications of the CARES project and at https://cares-project.eu. A visualisation of the database schematic is shown in Fig. 1 and a summary of the measurements captured across each campaign is presented in Table 1.

**Table 1:** Count of measurements from each city separated by instrument type: RS is optical remote sensing, PS is point sampling

City	Instrument	Count of measurements
Milan	PS	10852
	RS	35568
Prague	RS	120611
	PS	10658
Krakow	RS	128883
	PS	6162
**Total**	**All**	**1646031**

### Cloud computing platform

Microsoft Azure (https://azure.microsoft.com) provides Infrastructure as a Service (IaaS) through a range of virtual machine, storage and network offerings that allow a user to build and manage their own virtual infrastructure in the cloud. Azure’s Platform as a Service (PaaS) allows a user to develop and deploy web-applications, mobile applications and APIs without worrying about underlying infrastructure. PaaS is useful for building new applications quickly and without the need to worry about managing infrastructure. The data storage platform chosen for this project was CosmosDB (Guay Paz & Guay Paz, 2018).

CosmosDB is a globally distributed database service that allows for the creation and management of document, key-value, graph and column-family data models on one service. It is designed to offer high-throughput, low latency data access to applications around the world. The flexibility offered by CosmosDB is critical to the operation of the data platform. Data collected as part of remote emission sensing campaigns can be inconsistent from location to location and across instruments, with no official standard for the data being designated. As such, a data platform has to be flexible enough to accommodate significantly different data sets and serve them consistently to the end user for analysis. Data security is also an issue that CosmosDB solves effectively. As different data providers are subject to different data sharing agreements, and are not necessarily allowed to see each other’s data, using containers within the CosmosDB structure allows storage on the same database but limiting the access to designated containers depending on the case. Role based access can then be designed into the interfaces, limiting the availability of data to authorised user accounts. There is also a General Data Protection Regulation (GDPR, European Parliament and Council of the European Union (2016)) concern related to serving raw data to unverified end-users. Directly identifiable information such as vehicle registration number are not stored in the database, and vehicle passage time is truncated to the hour (a vehicle passing through at 15:27:00 will be registered at 15:00:00, for example). The truncation of the timestamp means that even with direct access to the database it would currently be impossible to regressively identify a vehicle and hence a user. A hashed and salted (Sriramya & Karthika, 2015) vehicle identifier is on occasion recorded, with the salt being changed regularly to avoid long-term vehicle usage trends being identified by bad actors. To guard against unanticipated future counter-security developments, only visualisations of aggregated and subset data are presented to the user. Neither the individual measurement data, or the tabular aggregated data are ever presented to the end user. The user may not directly query the database themselves without prior permission being granted. This means that a user may get the benefits of the aggregated data but does not risk the consequences of a GDPR breach.

The data structure used on the database was the key-pair JSON style document. This method allowed for flexible storage and retrieval of a wide number of different data sources. The data structure was designed to be transferrable between instruments and measurement campaigns to allow for easy querying of the database by the web-app. An example showing the most important key pairs is shown below. 
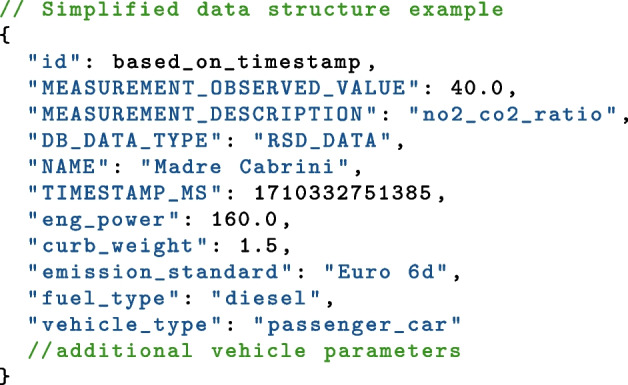


Data is accessed by querying the database using the NoSQL format. The web-app uses a range of widgets and inputs to build a query which is then sent to the database. The query is processed by the database and a data frame in a standardised format is returned to the web-app. The data frame is then converted into a plot. An example NoSQL query is shown below and would create a data frame containing two columns, one being the measurement value and one being the site name. The data returned would be all vehicles from the selected container (c) that match the emission standard, fuel type, vehicle type and measurement type. The CARES public app does not grant the user the ability to create custom text strings that can be sent as queries to both protect the database from malicious behaviour such as NoSQL injection attacks, and to preserve the standardisation of the plots that are generated. The secure science app, introduced in Section 2.3, allows for more customised analysis to be performed, including gross emitter vehicle candidate identification, point sampling plume time series analysis, and other in-depth questions. Users with access keys may build fully customised queries and analyse the data in their usual workflows.
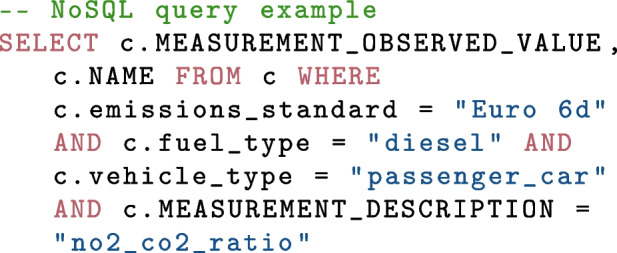


Measuring vehicle kinematics and emissions on the kerbside is a complex and often messy undertaking, with observations being influenced by external factors beyond the control of the measurer. Hence, quality control of the data is important especially when the end user may not be a technical expert. Various strategies are built into the data structure including the source file and data ingress time. This allows for rapid removal of the data as it is queried in the event of errors being discovered. Typically the measurements are left as delivered and adjustment factors are built into the post-process and data delivery stages of the infrastructure. A better conversion factor is more easily changed by adjusting a variable in the database query code than rebuilding the database from scratch. Additional technical quality control checks can also be applied as queries are built. For example, the vehicle specific power (VSP) in kW/T of a measurement is calculated as part of the RS measurement using speed and acceleration light gate measurements. Light gates have historically been shown to be very accurate (Rushton, 2016) however occasionally errors occur resulting in unphysical results. The observed VSP can be compared to the known power in kW and the vehicle mass in tons, to determine if the observed VSP was within the limits of the vehicle being measured. An example query is shown below:
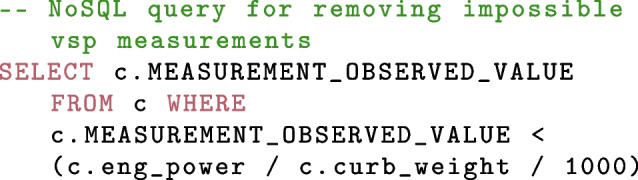


### Web-application

Web applications were developed to improve the accessibility of the data. Different users have different needs and so three web applications were developed to facilitate different levels of access to the data: the *City App* was developed for the core CARES team including government or city authorities of project partner cities and the *Science App* was developed for more experienced users of RS data to gain greater insight into the data. These two apps are protected by an authentication layer to further limit who may access the data, ensuring that all data sharing agreements remain intact. The third *Public App* was developed as a platform for dissemination of the project’s open data. This version is based on the *City App* and contains many of the same features but with access to data that is publicly available.

The web-application was developed in Python primarily using Streamlit (https://streamlit.io) as the front end as well as Altair (VanderPlas et al., 2018) and Pandas (Wes McKinney, 2010) to handle the data processing and visualisation. The web-app consists of a series of widgets designed to be user friendly that would build a set of queries to the database. The queries are then executed, returning an appropriate data set and the data set is then visualised in predetermined figures. This strategy allowed for the smoothest operation of the database and minimal cost due to data access. This also allows some quality control to be integrated by limiting exactly what analysis could be performed on the data.

The CARES *Public App* offers a wide range of high-level views on the data collected for each city within the CARES project. Table 2 shows the features available for each viewing mode.

**Table 2:** Summary of categories and subcategories of data visualisation available in the CARES *Public App*

Category	Subcategory
Fleet Composition	Euro Class
	Euro Class and Fuel Type
	Manufacturer
	Vehicle Type
Vehicle Dynamics	Vehicle Specific Power (VS)
	Speed and Acceleration
Vehicle Emissions	Emissions by Euro Class
	Emissions by Year of Registration
	Emissions by Manufacturer
Weather	Ambient Temperature
	Atmospheric Pressure
	Relative Humidity

Each of these modes may be viewed across the city as a whole or subset again by location. Each city has at least two different locations for data collection. Each measurement campaign has a different data specification and an objective of this project is to simplify the process of interacting with the data for the end user. To this end, the web app sorts through the user’s request and presents them with the appropriate data channels using natural language processing provided by the *fuzzywuzzy* python library (https://github.com/seatgeek/fuzzywuzzy). The full specification of each site is detailed in the CARES literature (Davison et al., 2022; Farren et al., 2023; Hao et al., 2022a, b; Knoll et al., 2023; Pechout et al., 2022; Pöhler et al., 2023; Qiu & Borken-Kleefeld, 2022; Yang et al., 2022).

## Outputs

This section will give a sense of the data visualisation capabilities of the CARES *Public App*. This web-app has been designed to be an interactive interface and whilst it is acknowledged that much of the value is lost when presented in a static format, it remains useful to demonstrate the capability where possible.

### Fleet composition

**Fig. 2:**
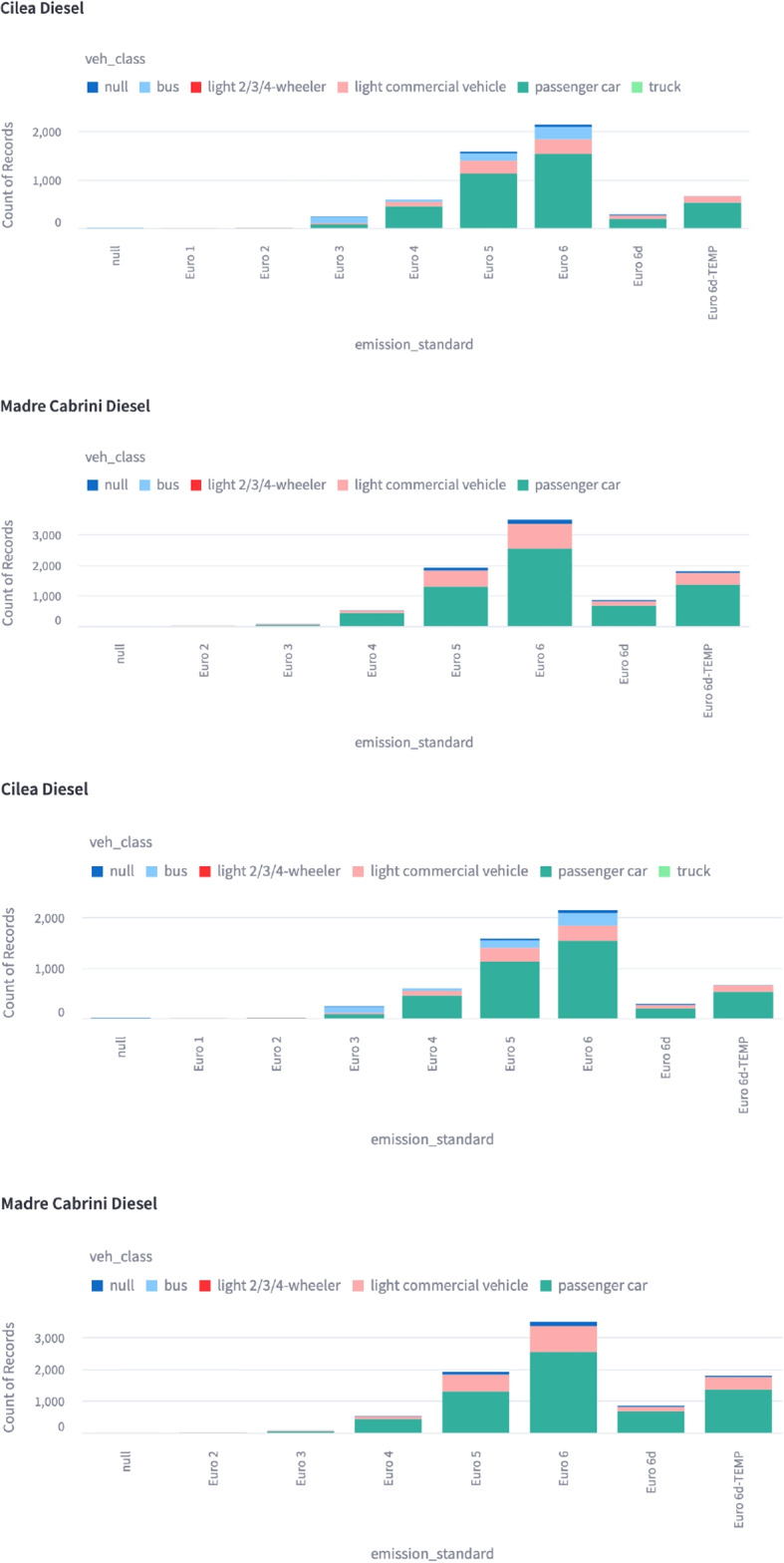
Figure showing the distribution of different diesel fuelled vehicle types (colour), emission standards (bars) and observation site (Cilea and Madre Cabrini facets) from the Milan campaign. A measurement recorded as null signifies that no metadata about a vehicle was available

Visualising the fleet composition is useful to understand the differences in the local fleet rather than relying on nationwide or super-national estimates of vehicle sales. Local classes of vehicles with high emission characteristics can be identified more readily and with greater confidence. Figure 2 shows how a user might see the different fleet subsets across two different sites, Cilea and Madre Cabrini (Milan). Some differences in the fleet composition is readily determined from Fig. 2 as Cilea has significantly more buses. This is useful information to have when designing policy as buses can be affected by different policies than passenger cars and private hire vehicles. There is also a higher fraction of newer Euro 6d and Euro 6d-temp vehicles in Madre Cabrini, suggesting different land use patterns and/or different demographics. Whilst some local knowledge might be assumed, the data presented allows for a direct reference to be made, free of assumptions about the underlying situation.

**Fig. 3:**
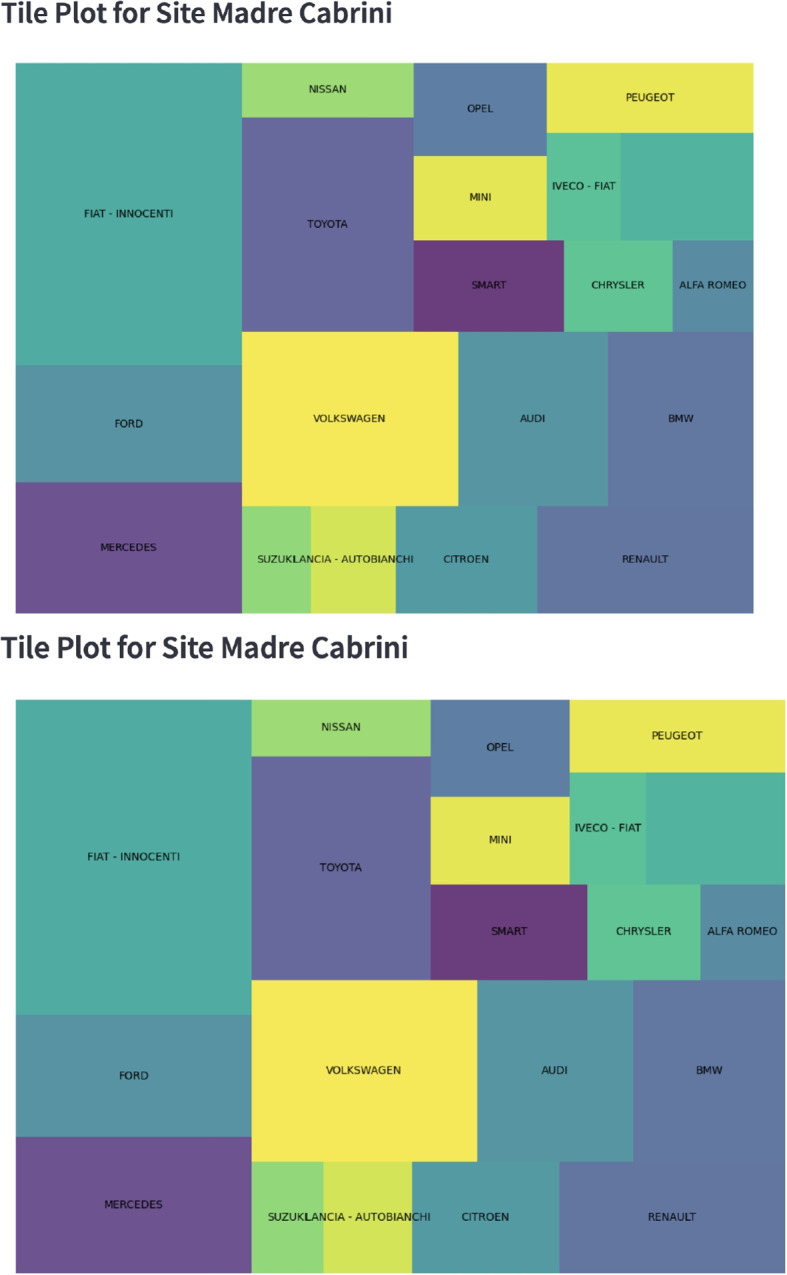
Web-app capture of figure showing the distribution of the vehicle fleet by manufacturer at Madre Cabrini, Milan, with a greater area indicating a greater number of records

It may be of interest to know the most prevalent manufacturers of vehicle that are present in a given location and the app and this is shown in Fig. 3. This information can be linked to vehicle emissions subset by manufacturers to identify any potential problematic trends.

### Vehicle emissions

**Fig. 4:**
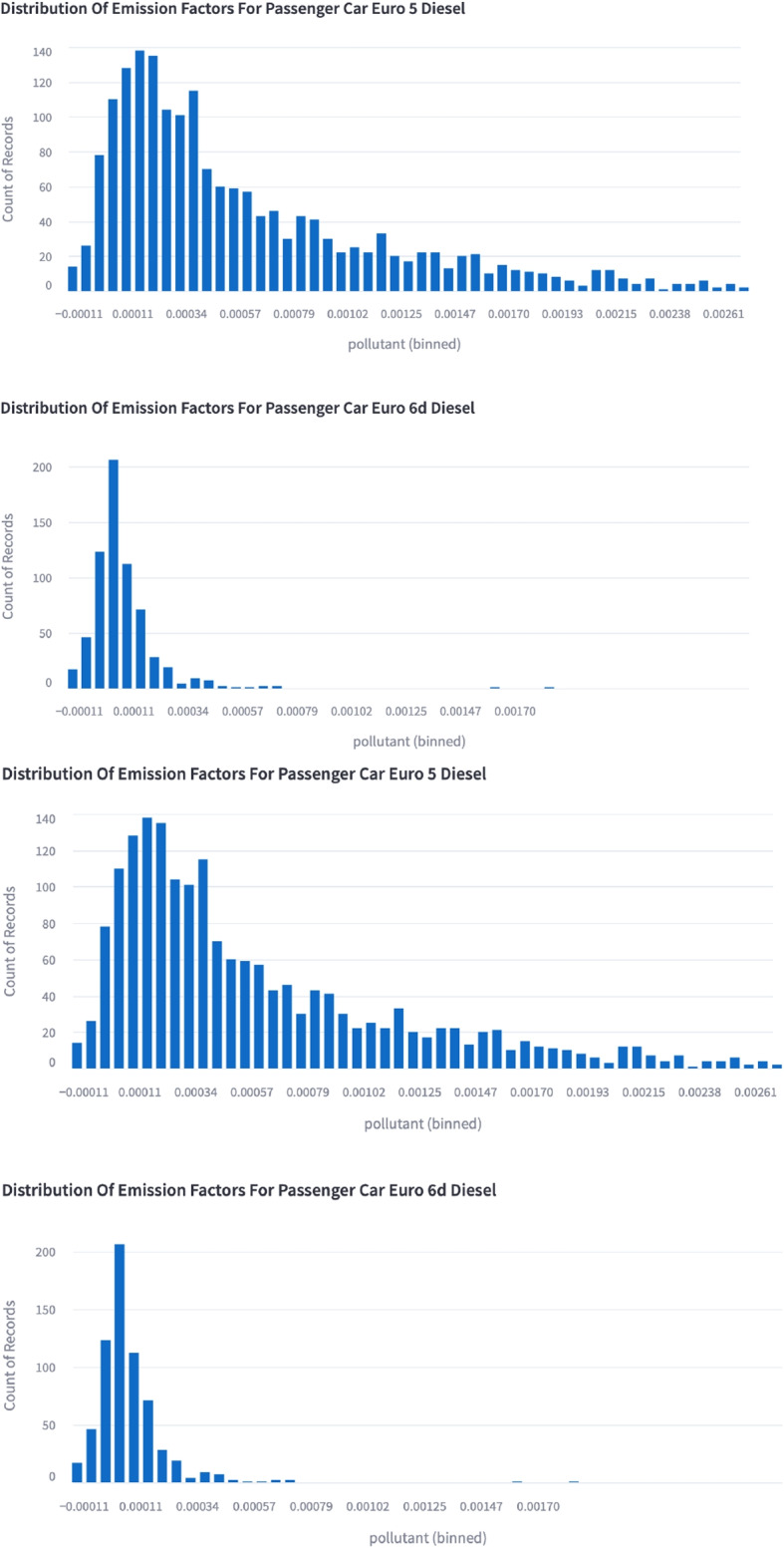
Web app capture of histogram showing the different distribution of emission ratios of $NO_{2} : CO_{2}$ from older Euro 5 diesel passenger cars and the newest Euro 6d diesel passenger cars taken from the Milan campaign

**Fig. 5:**
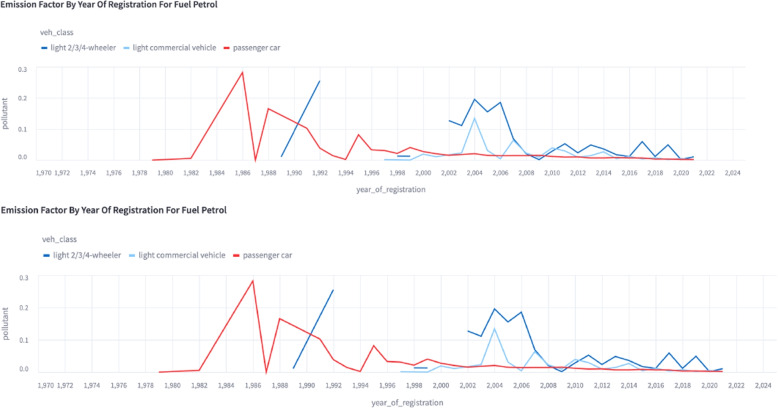
Web app capture of time series showing the magnitude of the mean $CO : CO_{2}$ emission ratios for each year of registration between 1970 and 2024 for petrol vehicles during the Milan campaign

Clean air zones do not always guarantee clean air (Holman et al., 2015) so understanding the emissions profiles of different vehicle subsets in a fleet is of critical importance to developing policy to reduce it. Various figures can be drawn using the *Public App* to help a policy maker understand the differences in RDE between different fleet subsets. Figure 4 shows two histograms of emission ratios of $NO_{2}:CO_{2}$ for two different fleet subsets. The top panel shows the older Euro 5 ($\approx$ 2009 - 2014) diesel passenger cars and the current Euro 6d (after date of first registration January 2021 - September 2024). The reduction in emission factors from Euro 5 to Euro 6d vehicles both in modal value and extreme values, as discussed in Yang et al. (2022) and Rushton et al. (2021) would indicate to the policy maker considering a low emission zone or clean air zone (CAZ) that allowing even the last generation of type approved vehicles in would reduce its impact quite significantly. A similar plot can be made using year of registration instead of Euro standard. This is useful when a longer time interval is being considered as it allows for the impact of deterioration to be visualised. Figure 5 shows the ratio of *CO* to $CO_2$ as a function of vehicle registration date. The ratio of *CO* to $CO_2$ for passenger vehicles is relatively flat after 2002, suggesting that passenger cars made after 2002 do not exhibit a significant change due to deterioration. Light 2/3/4 wheel vehicles remain stable after 2008, but show significant differences prior to 2008, suggesting that deterioration due to age is a significant factor in the overall emission ratio of these vehicles. The variance of light commercial vehicles is too high to identify a clear trend using this dataset.

**Fig. 6:**
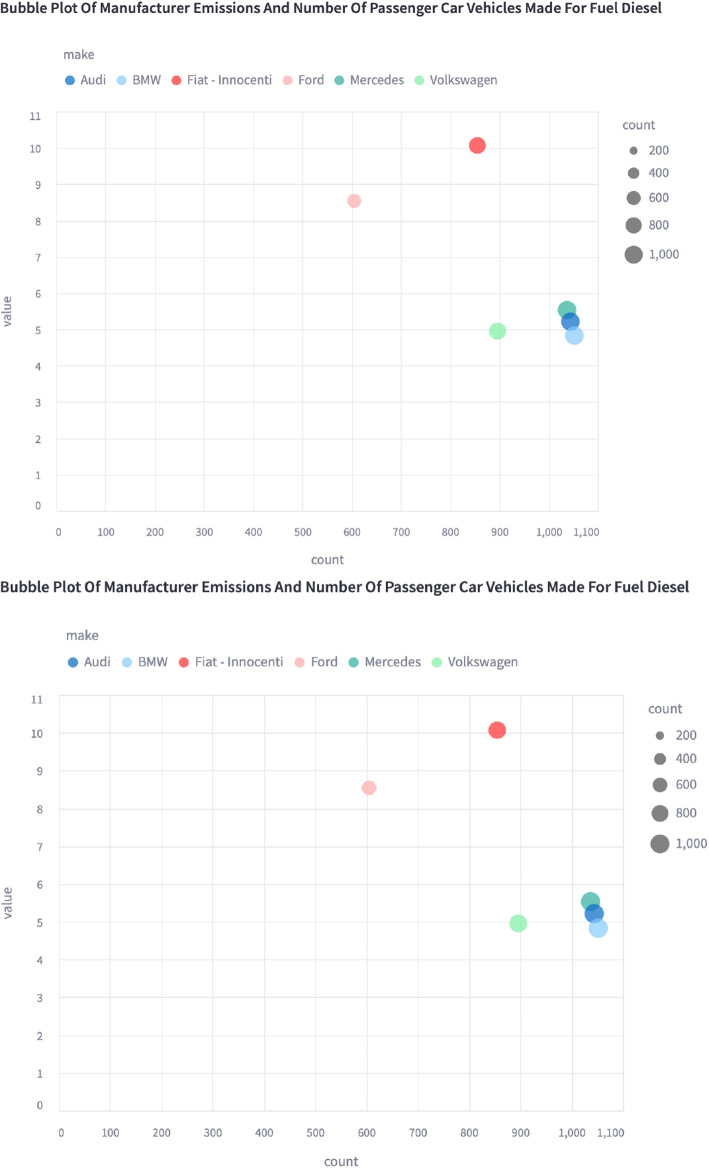
Web app capture of bubble plot showing the magnitude of the mean $NO_{X}$ fuel mass emission ratios and count of measurements for each of the six most prevalent vehicle manufacturers during the Milan campaign

The policy maker may also be interested to know if there are any systematic differences between different manufacturers in their fleet. Figure 6 shows how this might be visualised for the six most common vehicle manufacturers in the Milan campaign. This visualisation is helpful for identifying potentially problematic fleet subset but also putting them in the context of their prevalence. A small number of vehicles may be responsible for a large amount of the emissions (Rushton et al., 2021) and a more granular view of the fleet may help policy makers identify the most polluting vehicles and design policy to more effectively target them.

### Vehicle dynamics

Vehicle dynamics, usually presented as vehicle specific power (VSP) (Jimenez-Palacios, 1998) is an important metric for understanding vehicle emissions. $CO_2$ emissions increase linearly with increased power because of the increased physical work done. Air pollution emissions are often non-linear due to the complicated chemical reactions that underpin the catalytic processes in the pollution mitigation systems (Carslaw et al., 2013). Visualising the speed and acceleration characteristics of vehicles at a site gives operators a better understanding of the real driving conditions at a given site and will allow more specific policy to be developed. The measurable distributions of speed and acceleration, alongside local knowledge possessed by the government or city authority officers in charge can lead to much more effective policy development than relying on national averages or similar crude aggregate values.

### Weather

Ambient weather conditions (Fikeraddis & Endeshaw, 2020) are important for understanding the external influencing factors on air pollution concentration. Factors such as temperature, wind conditions and humidity influence the concentration and type of air pollution at a site and may be measured by some remote sensing instrument clusters. $NO_X$ and volatile organic compounds (VOC) interact in the presence of sunlight to produce photochemical smog (Dimitriades, 1972). Additionally, $NO_2$ emitted from tailpipes (primary $NO_2$) reacts in the presence of sunlight ($h\nu$) through photodissociation to create ozone ($O_3$) and the reaction of nitric oxide (*NO*) with $O_3$ creates secondary $NO_2$. Colder temperatures can also impact air pollution due to an increase in tailpipe emission due to cold starts and colder running (Dardiotis et al., 2013). It is important to understand the difference in context when determining the best approach for air pollution mitigation. Strategies that work in colder locations may not work in warmer ones, and policy makers need to be aware of these differences.

## Summary and future work

### Summary

The work presented in this paper is a timely and necessary advancement required for increasing the impact of direct measurements of vehicle emissions in-situ. The presented methodology for data organisation, analysis and dissemination represents a flexible and scalable solution to vehicle remote emission sensing data, which has so far been limited to paper documentation and static markdown documents. The potential of this approach has been recognised and the collective of researchers developing remote sensing technology, part of the ERMES group (https://ermesgroup.eu/web), have access to this platform and have been using it to inform their decision making and planning for future work.

Since the deployment of the CARES *Public App* there has been interest from additional authorities with their own data. These include data from campaigns conducted in Bosnia, Ireland and Switzerland. These data are yet to be added to the CARES *Public App* because they are covered by different data sharing agreements. In the future they may become available to the wider vehicle emissions and air pollution community, and the public more generally.

Remote sensing campaigns are still expensive to run and the data collected remains proprietary but with the expanded capabilities of the CARES *Public App* leading the way, it is probable that in the future there will be a shift in attitude towards a more open data approach. A more open data approach, coupled with the automation of standard remote sensing tasks within a secure environment and a more intuitive interface, would go a long way to standardising the processes and outputs from this research. The standardisation will facilitate further research including cross-site analysis and identification of synergies and differences across geographical locations. These new insights will ensure that the people responsible for making air quality policy decisions have the best possible tools and data available.

This work has successfully developed a data infrastructure that utilises cloud computing and data science for the analysis of vehicle emissions, demonstrating a pragmatic approach to environmental monitoring and policy support. Through the integration of traditional remote sensing and innovative point sampling methods, the project offers an enhanced framework for the detailed investigation of real-driving emissions (RDE) in urban settings. This infrastructure not only facilitates an improved evaluation of air pollution sources but also lowers the barrier of entry for policymakers and researchers to access and analyse real driving emissions data. Importantly, the data platform is publicly and freely available online, providing a valuable resource for ongoing and future environmental research and policy development, as well as citizen engagement. The collaborative nature of this project reflects a forward-thinking approach to addressing the complex challenges of urban air quality management. This work can be used as an example of leveraging technological advancements in the pursuit of creating sustainable urban environments and improving public health.

### Future work

Data will continue to be collected and measurement methodologies will continue to improve and diversify. A flexible and scalable approach to handling and serving this data to those that require it will continue to be important to ensure that the maximum impact is achieved from these measurements. There are four areas identified for future work to enable this platform to continue to make meaningful contributions to the vehicle emissions and air pollution policy discussion.

- Addition of new data from future remote sensing surveys
- Integration of supplementary third-party data sets
- Improve time-series data capacity
- Integration of IoT for close to real-time analysis

As more surveys are conducted using remote sensing technology, and increasingly using point sampling there will be an ever-increasing data resource that needs to be managed. The data structure of this project has been designed with this in mind and is flexible enough to handle most different data sets. Increasing the breadth and depth of the data set will allow for more and more reliable conclusions to be drawn. It will also enable policy to be more finely tuned to the context it will be deployed in. Currently, data is added to the database on a case-by-case basis and no automatic procedure has been developed. Data to be added is manually quality controlled and judgements about standardisation are made before adding it to the database. This process currently takes a week of development time depending on the similarity, size and complexity of the new data. It is beyond the scope of the work to develop an automated process at this time and researchers interested in engaging with this project are encouraged to contact the paper’s authors.

Currently inter-city comparison is not appropriate as the three cities presented represent very different contexts. Differences in fleet composition, climate, and city policy must be taken into account so analysis resembling like-for-like comparison may be misleading and self-defeating. As the data resource increases the opportunity to do more comparative analysis will present itself though. As more data from cities with similar contexts are made available the contextual variables can be controlled and tested using a range of different methodologies and the web-app platform will be able to present this analysis to a wide range of stakeholders.

Raw data from point sampling and also from mobile instruments deployed in plume chasing vehicles take the form of time series data. Time series data may not be used in the same way as snapshot data in the presented data structure. The CosmosDB document format allows for extended measurements to be included in single documents. A potential new data structure for these specific types of measurements may be introduced to the database to facilitate deeper understanding and more diverse data sets than simple snapshots.
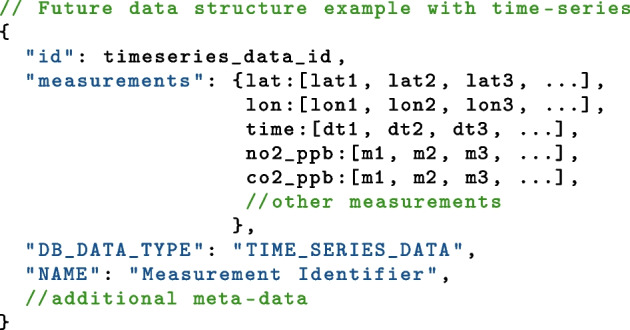


Supplementary data sets can currently be accessed in delayed time. Two data sets of interest to air pollution analysts might be the United Kingdom’s Automatic Urban and Rural Network (AURN) data set and the National Oceanic and Atmospheric Administration (NOAA) Integrated Surfaces Database (ISD) data set. These data sets are stored as a series of RDA data files compatible with the R programming language. Both of these can be accessed in R using the *openair* and *worldmet* packages respectively. They may also be accessed using Python using the *pyreadr* package (https://pypi.org/project/pyreadr/) and connecting directly to the data sources. The AURN dataset which can be accessed online is updated at around midnight each day and access to the previous day’s data is added. The NOAA ISD data set availability is delayed by two days. Neither of these data sets are therefore suitable for exactly real-time comparison but can be integrated into any longer-term analysis in an on-demand fashion.

The main RS technological developments moving forward are likely to be infrastructure based as well as scientific improvements. The current approach to data retrieval from the instruments remains slow and cumbersome despite efforts to improve speed in serving data to the final user. A further area for improvement is making the devices Internet of Things (IoT) capable. Once RS devices can connect to the cloud the devices could begin transferring emissions data to the database in real time. The Azure platform has full IoT functionality built in and connecting devices to the cloud would allow local authorities and policy developers the ability to see, in close to real-time, what is happening on their streets. GDPR regulations do not allow the storage of this data without significant modifications and removals of personally identifiable data. Whilst a public good argument might be made to improve access, the data collection infrastructure and processes would need to be significantly overhauled. This overhaul would include the addition of significant automated processes. Meta-data which is of interest to the scientific community as well as the regulatory community may not be as granular as historical remote sensing data, however demonstration of a secure IoT platform will increase confidence of data holders that RS data systems can be secure.

## Data Availability

The raw data for this project is not publicly available and neither is the source code for the app presented. These are to ensure compliance with any data protection rules that may apply, specifically GDPR. The front facing app is publicly and freely available at https://cares-public-app.azurewebsites.net.
